# Are you tripping comfortably? Investigating the relationship between harm reduction and the psychedelic experience

**DOI:** 10.1186/s12954-022-00662-0

**Published:** 2022-07-24

**Authors:** Madelene Palmer, Olivia M. Maynard

**Affiliations:** 1 School of Psychological Science, University of Bristol, Bristol, UK; 2 MRC Integrative Epidemiology Unit (IEU), University of Bristol, Bristol, UK

**Keywords:** Psychedelics, Harm reduction, Recreational drug use, Mixed methods

## Abstract

**Background:**

Alongside a recent revival in the use of psychedelics in clinical settings, there have been increases in the prevalence of recreational use, with many using psychedelics to deal with difficult emotions or to improve well-being. While clinical research is conducted in carefully controlled settings, this is not necessarily the case for recreational use. In this mixed methods online survey study, we aimed to develop an understanding of frequently used psychedelic harm reduction practices in recreational settings and how their use relates to the psychedelic experience. We also aimed to characterise users’ first and most recent psychedelic trips to understand how harm reduction changes with experience.

**Methods:**

Participants (*n* = 163) recounted their first and most recent psychedelic experience by providing details about the harm reduction practices they employed and completing the Challenging Experience Questionnaire (CEQ) and Emotional Breakthrough Inventory (EBI). We also asked open-ended questions for a more in-depth qualitative understanding of their views on psychedelic harm reduction.

**Results:**

Using ANOVA, we observe greater use of harm reduction practices for participants’ most recent versus first psychedelic experience and that use of these practices is positively associated with EBI scores and negatively associated with CEQ scores (particularly for the first experience). Participants engaged in a wide range of harm reduction practices and we provide details of those which are most commonly used and those which are deemed most important by experienced users. Our qualitative analysis indicated that participants were largely positive about psychedelics and many recounted profound positive experiences. While specifics of the drug they were taking was important for aspects of harm reduction, participants largely focused on the importance of ensuring a good “set and setting” for enhancing positive effects.

**Conclusions:**

Our research helps us understand how engagement in harm reduction may increase with experience. Our mixed methods data shed light on the perceived importance of different harm reduction practices and examine their association with the psychedelic experience itself. Together, our research has important implications for the development of psychedelic harm reduction advice and provides opportunities for future research to explore the importance of these different practices in more detail.

**Supplementary Information:**

The online version contains supplementary material available at 10.1186/s12954-022-00662-0.

There has been a recent revival in clinical research examining the effects of psychedelics in treating a wide range of conditions [1, 2]. At the same time, among those who self-identify as drug users, there is evidence of an increase in the use of psychedelics in recreational settings [3] (although this increase is not seen among the general population [4]). The 2021 Global Drugs Survey [3] found that among recreational drug users between 2015 and 2020, use of LSD increased from 9 to 21%, psilocybin from 9 to 16% and DMT from < 1 to 5%. The majority (52%) of those using psychedelics reported doing so to improve their well-being, with others reporting use to deal with emotional concerns (32%) or to address a psychiatric condition (15%). Other evidence suggests psychedelics are used recreationally to attempt to self-treat anything from eating disorders [5], to racial trauma [6], chronic pain [7] and colour blindness [8]. This suggests that recreational users are attempting to benefit from the therapeutic effects of psychedelics observed in clinical studies in recreational settings and applying them to a wide range of untested issues. There is some [9], but not consistent [10] evidence that past-year use of psychedelics is associated with depression and suicidal thinking, and although the direction of this relationship is unknown, this finding may support the view that individuals suffering from these conditions are drawn towards trying psychedelics in an attempt to self-treat.

Clinical psychedelic studies are conducted in carefully controlled environments where few adverse events have been reported [2]. There is considerable literature providing guidelines for safety in clinical settings [11], including advice for professional psychedelic therapists [12, 13]. However, there is scant research about reducing harm for recreational users. Previous research has examined harm reduction practices used by those microdosing psychedelics (i.e. routinely taking small amounts of psychedelics) [14], but not for those taking larger doses. While drug researchers [15] and experienced users [16] report that the risks of recreational psychedelic use are low, and negative psychological consequences are uncommon and generally mild, there can be acute and, in some cases, longer term negative effects [17]. Carbonaro and colleagues examined psychedelic users’ most difficult experience (i.e. a “bad trip”) [18], with 24% reporting negative psychological symptoms lasting one week or more, including fear, anxiety, depression and paranoia, with 10% reporting negative symptoms a year afterwards. For most people, however, these difficult experiences were not wholly negative, with 84% reporting benefitting from the experience, with the degree of difficulty positively correlating with personal meaningfulness. It is unknown, however, whether these positive effects were because of, or despite, the difficult experience.

Carthart-Harris and colleagues [19] suggest that the “therapeutic action of psychedelics is fundamentally reliant on context”, and that neglecting either the psychological (i.e. set) or environmental (i.e. setting) context may reduce their effectiveness, or even cause harm (see Hartogsohn [20] for a review of set and setting). Without the careful planning and psychological support available in a clinical study, those using psychedelics recreationally may be at greater risk of poor set and setting, possibly increasing the risk of difficult experiences. Psychedelic harm reduction must focus on how to reduce these psychological harms, and given users’ motivations for use, maximise the possibility of positive effects. Previous qualitative research has examined the harm reduction practices experienced users employ before, during and after a psychedelic experience [21] and the factors reported to be associated with both positive and negative experiences [22], but no quantitative research has examined the frequency or perceived importance of these practices, or the extent to which their use is related to the psychedelic experience.

It is possible that more experienced psychedelic users (such as those in the Carbonaro study described above) may employ more harm reduction practices and may be better equipped to deal with challenging experiences. Indeed, in their qualitative interviews with experienced psychedelic users, Gashi and colleagues [23] find that while most users reported having had frightening experiences, they also reported mitigating their effects by respecting set and setting, such that bad trips were generally perceived to be due to an “immature and irresponsible” approach to psychedelics [23]. They also found that experienced users engaged in “post-trip meaning making” to construct narratives transforming frightening or unpleasant experiences into positive ones. Given this, it is possible that difficult experiences may be more likely for inexperienced users, due to poor awareness of the importance of harm reduction methods promoting a positive set and setting, as well as fewer opportunities to discuss the experiences with other psychedelic users. To our knowledge, no previous research has compared the experiences of or methods of promoting positive set and setting among first time compared with experienced psychedelic users.

As such, we aimed to first, support recommendations for safer, more positive psychedelic experiences, by understanding the extent to which different harm reduction practices are deemed effective for a safe trip by experienced psychedelic users. Second, we aimed to understand how engagement in harm reduction practices changes with experience, by comparing users’ first and most recent psychedelic trips, hypothesising that people would engage in more harm reduction practices perceived as effective for their most recent trip. Finally, we examined the relationship between harm reduction practices and the psychedelic experience; hypothesising that experiences where more practices perceived as effective are used will be positively associated with emotional breakthrough and negatively associated with challenging experiences.

## Methods

### Design

This online study used an observational, repeated-measured survey design with psychedelic users providing details of their first and most recent psychedelic experiences. We assessed use of a range of harm reduction practices as well as the prevalence of challenging experiences and emotional breakthrough. We pre-registered the study on the Open Science Framework (https://osf.io/35kbs/; https://doi.org/10.17605/OSF.IO/35KBS).

### Participants

Participants were recruited opportunistically through relevant university societies and online psychedelic communities. Eligible participants were required to be aged 18 or over, have consumed a classic psychedelic (i.e. LSD, psilocybin, DMT, mescaline) or an analogue or derivative of a classic psychedelic (e.g. 2C-B) at least twice in their life and be fluent in English (see Supplementary Materials for details of participant exclusions). Neither participants’ first nor most recent psychedelic experience could have been in a non-recreational setting (i.e. a controlled research study, or professional therapeutic setting) nor could they have microdosed the substances on either occasion (given the lack of consensus on what constitutes a microdose, no definition was given and participants determined for themselves whether the dose they had taken was more than a microdose).

### Measures

#### Demographics and psychedelics use

Participants reported their age, gender and level of education as well as the number of times they have used a psychedelic substance and the psychedelics they have previously used.

#### Characteristics of first and most recent psychedelic experiences

For both their first and most recent psychedelic experiences, participants reported the time (in months and years) since their experience and the substance(s) they used (from a list including LSD, psilocybin, DMT, mescaline, 2C-B and “other”). Participants also reported the setting they consumed the substance(s) and their intention/reasoning for using (more details in Table 1).

#### Harm reduction practices

We developed a list of recommended harm reduction practices by searching trusted online harm reduction resources and the published literature, as well as through discussion with colleagues (see Additional file 1: Table 3 Supplementary Materials for a list of practices selected and the sources searched). We split these practices into those performed before, during and after the psychedelic experience. Within the “during” section we included some practices which are either common anecdotal myths (e.g. drinking orange juice will reduce the chance of a “bad trip”), or those which are generally recommended against, at least for inexperienced users (e.g. being alone in a dark space) to explore participants’ use of and perceptions of these, but also as a method of encouraging participants to think carefully about the list.

Participants selected the practices they used for their first and then most recent psychedelic experience. If they did not use any of the practices, they could select “none of the above”. Participants also rated how important they thought each strategy was for a safe experience with the following anchors: -10 “detrimental to a safe psychedelic experience”; 0 “does not affect the safety of a psychedelic experience”; 10 “extremely helpful for a safe psychedelic experience”. We calculated a Harm Reduction Score for each practice by taking a mean of participants’ scores for that practice. We calculated individuals’ Participant Harm Reduction Score by summing the Harm Reduction Scores for the practices they reported using for each of their first and most recent experiences. Due to a methodological error, the practice “Arranged a Time to take the dose” did not have any scores available.

#### Challenging Experience Questionnaire (CEQ)

The CEQ was developed using an Internet survey of those reporting a wide range of challenging experiences while using psilocybin and has been shown to have good internal consistency and external reliability [24]. It consists of 26-items forming seven subscales: Isolation, Grief, Physical Distress, Fear, Insanity, Paranoia and Death. Participants rated the extent to which they experienced each of the phenomena on a 0–5 scale (with 0 being “None, not at all” and 5 being “Extreme, more than ever before in my life”). Scores for the subscales were calculated by taking a mean of the items they comprised. Due to an experimenter error, two of the three questions from the Insanity subscale were not asked: “I experienced a decrease sense of sanity” and “I was afraid that the state I was in would last forever” and this subscale therefore comprised only one item. All scores were presented as a percentage of the total score available.

#### Emotional Breakthrough Inventory (EBI)

The EBI was developed using an Internet survey of those who reported using a psychedelic. It is a reliable and validated scale which is positively associated with increases in well-being after a psychedelic experience [25]. The EBI consists of six statements such as “I felt able to explore challenging emotions and memories” and asks about “emotional release”, “closure”, “emotional breakthrough” and “resolution of conflict”. Participants rated the extent to which they agreed with each statement on a 0–100 scale (with 0 being “No, not more than usually” and 100 being “Yes, entirely or completely”).

#### Attention checks

To ensure engagement with the study, there were three attention checks (details in Supplementary Materials).

### Procedure

Online advertisements directed prospective participants to the study hosted on Qualtrics (www.qualtrics.com). Following informed consent, eligible participants completed the demographic questions. Participants recalled their first psychedelic experience and completed the questions related to the characteristics of this experience. They then reported which harm reduction approaches they used and completed the CEQ and the EBI. Participants then repeated this for their most recent psychedelic experience. Participants reported how important for a safe experience they thought the harm reduction approaches were. Finally, participants provided feedback through a free-text entry box before being shown the debrief form. Participants had an option to follow an additional link to enter a £50 voucher prize draw.

### Statistical analysis

We used JASP to analyse the data. Our data analysis plan follows that described in our pre-registered protocol and we highlight any exploratory analyses. Participants provided considerable information in the free-text response box, and we used thematic analysis to identify and interpret patterns across this qualitative dataset and provide a more detailed understanding of participants’ harm reduction practices. We based our approach on methods which have previously been used to interpret free-text survey responses [26, 27]. Data analysis was primarily conducted by the last author, in collaboration with the first author. Through discussion throughout, both authors reviewed the data, the coding process and theme generation. We coded 61 free-text responses to the open-ended question. We completed a three-stage process of inductive analysis 1) coding of the entire dataset, 2) development of initial themes and 3) refinement of initial themes after discussion of the “narrative” of the themes and by revisiting the whole dataset.

## Results

### Participant characteristics and psychedelic use history

There were 163 participants after exclusions (see Supplementary Materials for details). As shown in Additional file 1: Table 1, the mean age of participants was 27.5 (SD = 8.06) although the mode [20] and median [26] age were lower (range 18 to 56), due to a large number of younger participants and a small number of participants (*n* = 13) aged 40 or above. There were considerably more male (*n* = 120) than female (*n* = 35) or non-binary (*n* = 8) participants. When asked what psychedelic drugs they had previously taken, participants reported broad experience, with LSD and psilocybin being the most common. Participants estimated having used psychedelic drugs a mean of 28.4 times (SD = 45.2). This mean was highly skewed due to a small number of considerably higher use estimates (300 uses *n* = 2; 200 uses *n* = 2; 150 uses *n* = 2; 100 uses *n* = 10). The mode [20] and median (11.5) use estimates were considerably lower.

### Characteristics of first and most recent psychedelic experiences

Table 1 presents the characteristics of participants’ first and most recent psychedelic experiences. Given the wide variability in these estimates, we report the mean, mode and median time since these two experiences. This variability was largely driven by a small number of older participants who had been using psychedelic drugs for decades. Given that the mode age of participants was 20, it is perhaps unsurprising that the mode time since first use was two years.

**Table 1:** Characteristics of first and most recent psychedelic experiences

	First experience (*n* = 163)	Most recent experience (*n* = 163)
How long ago in months (mean)	67.52 (78.33)	8.68 (20.76)
How long ago in months (mode)	24	1
How long ago in months (median)	42	2
Drug used n (%)		
LSD	76 (47)	54 (33)
Psilocybin	67 (41)	58 (36)
2C-B	6 (4)	9 (6)
DMT	3 (2)	10 (6)
Mescaline	1 (1)	2 (1)
Other (see Supplementary Materials)	10 (6)	30 (18)
Location n (%)		
At home *	67 (41)	93 (57)
At a trusted friend’s house	51 (31)	29 (18)
In nature	18 (11)	19 (12)
At a festival	11 (7)	6 (4)
At a club	8 (5)	8 (5)
In a ceremonial setting	0 (0)	4 (2)
Other		
House party	3 (2)	0 (0)
Outdoor party (rave, solstice)	2 (1)	0 (0)
Not clear (e.g. name of city given)	2 (1)	4 (2)
At school	1 (1)	0 (0)
Intention n (%)^		
Out of curiosity	118 (72)	32 (20)
For fun/recreationally	107 (66)	93 (57)
For spiritual pursuits	32 (20)	75 (46)
To find a sense of self-enlightenment	31 (19)	67 (41)
To explore the therapeutic properties	24 (15)	61 (37)
To process difficult emotions	15 (9)	52 (32)
For artistic inspiration	15 (9)	40 (25)
I did not have an intention/reasoning	6 (4)	1 (1)
Other (see Supplementary Materials)	4 (2)	10 (6)

^*^ Where participants report being in two places (e.g. at home and then in nature), we have reported this as the first place mentioned. “At home” also refers to temporary residences, such as hotels, holiday cottages and student dormitories

^ Participants could select more than one intention

LSD and psilocybin were the most commonly reported psychedelics used in both first and most recent experiences, although a wide range of psychedelics were used, with some participants reporting polydrug use (with other psychedelics or other non-psychedelic drugs).

Most participants reported using psychedelics at home, with more reporting home use for their most recent experience (first = 41% most recent n = 57%). The second most popular location was at a trusted friend’s house, with more participants reporting this location for their first experience (first = 31%; most recent = 18%).

Participants’ intentions were different for the two psychedelic use experiences. The most common intentions for the first experience were curiosity and fun. While fun was still a commonly cited intention for the most recent experiences, other intentions such as spiritual, self-enlightenment, artistic, therapeutic and processing of emotions were more common for the most recent experience.

### Harm reduction practices used during first and most recent psychedelic experiences

**Fig. 1:**
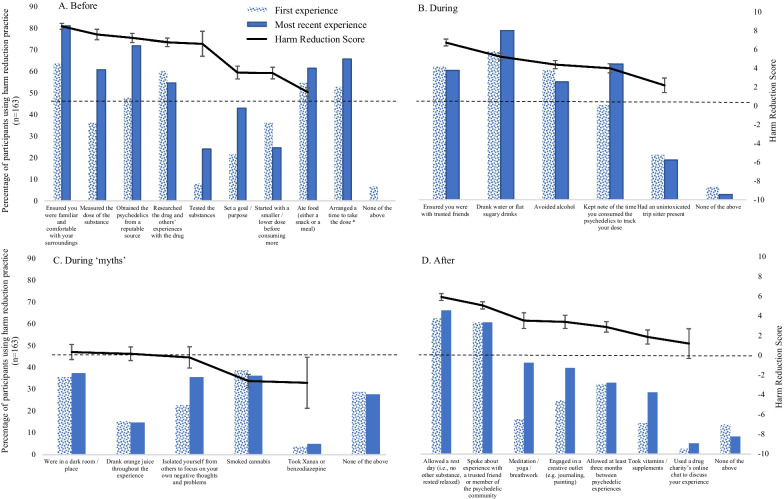
Prevalence of use for each harm reduction practice (bars) and Harm Reduction Scores (line; -10 “detrimental to a safe psychedelic experience”; 0 “does not affect the safety of a psychedelic experience”; 10 “extremely helpful for a safe psychedelic experience”. Error bars represent standard errors

Figure 1 shows the percentage of participants who used each of the listed harm reduction practices for their first and most recent experiences. The line on the secondary axis shows the mean Harm Reduction Scores for each practice.

To examine the difference in Participant Harm Reduction Scores between experiences, we ran a repeated measures ANOVA of time (before, during, during-myths, after) and occasion (first, most recent). This revealed a main effect of occasion (*F* (1,162) = 28.32, *p* < 0.001, *h*_*p*_^2^ = 0.012), indicating that, as hypothesised, Participant Harm Reduction Scores were higher for their most recent experience (*F* (3, 486) = 563.82, *p* < 0.001, *h*_*p*_^2^ = 0.78). We also observed an occasion × time interaction (*F* (3,162) = 24.76, *p* < 0.001, *h*_*p*_^2^ = 0.133), and Bonferroni corrected comparisons indicated that Participant Harm Reduction Scores were higher for the most recent psychedelic (compared to first) experience for the approaches used before (*t* = − 9.63, *p* < 0.001) and after (*t* = − 3.28, *p* = 0.03), but not those used during (*t* = − 1.47, *p* = 1) or during-myths (*t* = − 0.04, *p* = 1). The main effect of time is not meaningful given the items which comprise these scales are not comparable.

### Experiences of first and most recent psychedelic experiences

We observed high levels of internal reliability for the Challenging Experience Questionnaire (CEQ) and Emotional Breakthrough Inventory (EBI) scales (CEQ first experience: Cronbach’s alpha *α* = 0.96; CEQ most recent experience: *α* = 0.95; EBI first: *α* = 0.92; EBI most recent: *α* = 0.93). Table 2 shows the mean scores (SD) for the EBI and each of the subscales of the CEQ. The mean CEQ score of 17 for both first and most recent experience is in line with previous research [25]. Exploratory analyses indicated that EBI and CEQ scores were positively correlated for both first (*r* = 0.393, *p* < 0.001) and most recent experiences (*r* = 0.369, *p* < 0.001). A repeated measures ANOVA with each of the seven CEQ subscales and the two occasions as the two factors demonstrated that there were no meaningful differences between subscale scores for first and most recent experiences. A paired samples *t* test indicated that the EBI scores for the most recent experience were meaningfully higher than the first experience (*t* (162) = 4.42, *p* < 0.001, *d* = 0.35).

**Table 2:** Harm Reduction Scores, EBI and CEQ for all participants and split by those who reported using the psychedelic in party as compared with other settings

	First experience			Most recent experience		
	All participants (*n* = 163)	Party setting (*n* = 24)	Other setting (*n* = 139)	All participants (*n* = 163)	Party setting (*n* = 12)	Other setting (*n* = 151)
Harm Reduction Score	40.06 (18.90)	21.95 (12.61)	43.20 (18.57)	48.68 (18.81)	26.23 (12.25)	50.46 (18.10)
EBI	27.77 (26.46)	20.38 (24.16)	29.05 (26.71)	39.18 (29.91)	41.56 (23.56)	39.00 (30.31)
CEQ	17.14 (18.06)	19.27 (18.02)	16.77 (18.10)	17.29 (17.06)	31.46 (24.02)	16.16 (15.96)
Isolation	19.14 (25.14)	29.44 (29.35)	17.36 (24.02)	18.24 (22.29)	33.89 (26.28)	16.99 (21.56)
Grief	15.52 (20.99)	17.36 (21.24)	15.20 (21.00)	19.12 (21.68)	28.33 (25.21)	18.39 (21.30)
Physical distress	20.69 (19.68)	21.33 (18.93)	20.58 (19.87)	20.54 (20.56)	39.67 (22.85)	19.02 (19.66)
Fear	19.07 (24.54)	20.83 (25.76)	18.76 (24.41)	16.59 (22.54)	29.67 (26.45)	15.50 (21.97)
Insanity	17.30 (26.81)	21.67 (32.26)	16.55 (25.81)	11.90 (21.85)	30.00 (27.63)	10.46 (20.76)
Paranoia	9.88 (18.46)	10.00 (17.94)	9.86 (18.61)	8.77 (18.18)	30.00 (30.15)	7.09 (15.86)
Death	12.45 (24.14)	8.75 (19.18)	13.09 (24.90)	15.15 (24.33)	23.33 (25.70)	14.50 (24.19)

*EBI* Emotional Breakthrough Inventory, *CEQ* Challenging Experience Questionnaire

Values represent mean (standard deviation)

As hypothesised, there was evidence that Participant Harm Reduction Scores were positively correlated with EBI score (both: *r* = 0.24, *p* = 0.002) and negatively correlated with the first CEQ score (*r* = − 0.21, *p* = 0.006). Although in the hypothesised direction, there was no statistical evidence that these scores were correlated with the most recent CEQ score (*r* = − 0.15, *p* = 0.061).

Given the importance of setting in the psychedelic experience, in unplanned exploratory analyses we compared experiences and harm reduction approaches between those who took the psychedelic at a party/club/festival (hereafter “party setting” and those who took them in other settings (e.g. home, nature, trusted friends’ house, or other; hereafter “other setting”). The majority of participants did not take the psychedelic in party settings and this was even less common for the most recent experience (first *n* = 24; most recent *n* = 12). As there was a large difference in sample size and because these analyses were unplanned, we have not run statistical tests on the data, but instead we report descriptive statistics (see Table 2). As would be expected, Participant Harm Reduction Scores were lower when used in party settings. The CEQ score was higher for party settings (particularly for the most recent experience, although note the small sample size). For interest, we have also split the CEQ into its seven subscales—it appears that higher CEQ scores for party settings for first use were characterised by feelings of isolation. For the most recent experience, scores for all subscales were numerically higher. EBI scores were lower in party settings for first experience, but not most recent.

### Qualitative analysis of free-text responses

We have developed three broad themes which encapsulate participants’ responses to the free-text response box. These relate to 1) the profound positive experiences the psychedelics elicited, 2) the importance of the drug and 3) the importance of set and setting. Below we provide an analysis of these themes, alongside illustrative quotations (for longer quotations we have provided participant sex, age and the number of times they report having used psychedelics). Where appropriate we bring in evidence from the quantitative analysis.

#### Theme 1 Profound positive experiences

Participants frequently described profound psychedelic experiences which had “changed [their] life”. These experiences helped them feel “centered and connected with everyone and everything”, “closer to nature”, better able to “understand the nature of [their] mind” and allowed them to “come to terms with anxiety, depression, and nicotine addiction” or deal with “severe mental illness” “without the bad side effects that come with prescription psychiatric medications”. Participants felt that psychedelics “could help others like [them] if used in a professional therapeutic setting” as it would “open so many people's eyes to the universe and unconditional love”. Psychedelics were described as “super important medicine”.

Participants felt that the survey was limited in its scope as it failed to ask about these positive experiences. While the harm reduction practices largely focused on ways to reduce harm and asked about “safe” experiences, participants felt that it “missed some of the benefits, or things that might be done to enhance the experience” and how not all challenging experiences (i.e. the CEQ) were negative:

“not every hard trip is a ‘bad trip’. Sometimes you need to struggle to grow stronger” and that these “bad parts…make it worthwhile in the right amount” (male, 22, 5 uses).

#### Theme 2 Importance of the drug

Participants explained how the harm reduction practices they would use would vary depending on the psychedelic drug they were taking. While some commented on practices which, to our knowledge, are not based on scientific evidence (e.g. cannabis being “very negatively impactful on LSD” but “helpful when consuming psilocybin”, or fasting before 2C-B, but eating before LSD or psilocybin), others highlighted differences between psychedelics which make intuitive sense (e.g. testing being “super important” if using “pills/powder bought from an unknown source”, but less important for psilocybin in the form of mushrooms, particularly for those “one grew oneself”).

Indeed, testing the psychedelic before use (both to identify the substance and the dose) was a harm reduction practice which garnered considerable comments with participants reporting that if from a “trusted source”, testing was not important. The quantitative data suggest that while both practices received high Harm Reduction Scores, few participants tested their drugs, but many obtained their drugs from a reputable source, particularly for the most recent experience. Participants described the dose as “surely one of the most important factors for a safe psychedelic experience”. While some psychedelic doses can be easily measured, others, such as “LSD in tab form cannot be easily measured at home”. Participants reported that “starting with a smaller dose” and then having a larger dose the next time (as opposed to redosing within a single session) is “a good harm reduction practice”. The dose consumed and other components of set and setting were important factors underlying the choice to engage in harm reduction practices:

“I wouldn't do many of the harm reduction activities if I were taking a low dose, with friends, in a familiar setting. But if I were taking a very high dose (as I sometimes do in a solo therapeutic setting), then I'll do loads of harm reduction.” (male, 40, 40 uses).

#### Theme 3 Importance of set and setting

“Set and setting” were inextricably linked, such that a positive setting ensured a positive set, and these were referred to by participants either explicitly, or by describing the actions they take to ensure these were optimal for their experience. These actions were largely around creating safe, calm spaces and reducing stress and anxiety. Participants took set and setting seriously:

“I have rules when I trip. I don’t trip unless I know for a fact I have nothing to do the next day, I won’t trip if I’m already tired, stressed out, or depressed. I make sure I have music playing, and that I can be left alone” (male, 18, 40 uses).

##### Subtheme 3.1 Set and setting to improve positive experiences

Participants suggested that the actions they take to improve their set and setting largely were focused on improving the psychedelic experience, as opposed to reducing harm:

“psychedelics aren't half as scary as most make them out to be. As long as your set and setting is good…it can be a real enlightening experience” (male, 19, 45 uses)

Indeed, many participants distinguished between practices which reduced harms and those which increased the likelihood of positive effects, with one participant noting that many of the practices “didn't affect the safety of the psychedelic experience, [they did] affect the experience, just not the safety of it” (female, 25, 300 uses).

Participants felt that one of the harm reduction practices listed in the survey should have been “making sure you are in a good mood before tripping”. Participants talked about the importance of clearing their minds before a psychedelic experience, through meditation or mindfulness practices. Others described how it is “essential” to make “sure that you have no prior commitments on the day of the trip…[and] limit the amount of time you are looking at devices (specifically your phone + social media) before and during a trip” (male, 19, 6 uses).

##### Subtheme 3.2 Darkness versus light

One of our harm reduction “myths”, which is advised against on multiple harm reduction websites was “being in a dark room/place”. Approximately 35% of participants reported being in a dark room and this practice received a Harm Reduction Score close to zero, indicating that it was neither deemed beneficial nor detrimental to a safe experience. Participants highlighted that for this approach, the effects depend on other aspects of set and setting, such that it could be “scary if you feel alone” or “at a club this could be disorienting”, with one participant describing how this approach is a good example of how “not all harm reduction techniques affect everyone the same way. For example, some people may want to sit in a dark room and some people may not” (male, 18, 8 uses). Indeed, other participants noted that “being in a dark room can enhance the trip” or “can be good if it’s too intense” (male, 39, 200 uses).

##### Subtheme 3.3 Tripping alone versus in company

Tripping alone is also advised against on many drug harm reduction websites, although participants were ambivalent about this. Participants described a variety of different preferred scenarios: tripping alone, tripping with a sober friend, tripping with a sober guide and tripping with trusted co-trippers. Our quantitative analysis indicated that the majority of participants reported that they were with trusted friends for both experiences and this practice received a high Harm Reduction Score, while few participants reported having an unintoxicated trip sitter present and this practice received a low Harm Reduction Score.

Participants acknowledged that different approaches would be required for different people, based both on their experience and the type of trip they were planning. For example, “people with more experience might have no need for a tripsitter, while…it’s absolutely necessary for someone trying the first time”. Indeed, one participant described their first psychedelic trip which taught them that “harm reduction practices are extremely important” and the “really bad experience” they had “could have been avoided [by having] an unintoxicated tripsitter, safe space, music playlist”. Others suggested that having a sober trip sitter can be unhelpful as “being in different consciousness states makes communicating harder and can lead to “bad vibes” or “bad situations”. Many participants described the importance of being with “knowledgeable people you trust deeply, who will support your journey and who will keep you safe from harm” (male, 24, 8 uses). Others described the importance of a guide, and.

“heavily advise that people considering it for therapy and have no experience to do it in a professional setting such as a qualified psychedelic retreat when possible, or at least to have it with someone who has experience. Avoiding thoughts of an isolating negative nature is also recommended by checking them with the trip sitter as often times they would be exaggerated or not true due to tripping”. (male, 20, 12 uses).

## Discussion

Our cross-sectional survey provides the first detailed characterisation of experienced psychedelic users’ first and most recent psychedelic experiences, including the drugs they use, the harm reduction practices they employ and their experiences of use. Here, we discuss the results in the context of recommendations for those providing harm reduction advice for psychedelic users, with a focus on advice for first-time users.

Our qualitative data suggest that our self-selecting sample of experienced users were familiar with using harm reduction practices and they felt that ensuring appropriate set and setting was critical to the psychedelic experience. Harm reduction focused largely on creating safe, calm spaces and reducing stress and anxiety before, during and after the experience. As hypothesised, we find that engaging in these harm reduction practices was positively associated with emotional breakthrough for both the first and most recent experience, and negatively associated with challenging experiences (although this correlation was weaker for the most recent experience). This provides some preliminary evidence that harm reduction practices may reduce negative and enhance positive experiences. This supports previous research which finds that both CEQ and EBI scores can be predicted by the degree to which individuals endorse baseline ratings of therapeutic intentions, the creation of therapeutic settings and being in a mindset where they are willing to confront difficult emotions [25, 28]. These findings also lend credibility to our list of harm reduction practices which were rated for their importance for a safe experience by our participants, although future research should refine this list of practices further, as our mixed methods data suggest that not all practices were deemed important, or commonly used and the qualitative data suggested that other practices should be included.

All our experienced psychedelic users were first-time users once, and our data provide insight into how their harm reduction practices and their psychedelic experiences have changed over time. As hypothesised, we find greater use of harm reduction practices for participants’ most recent as compared with first experience, including ensuring they were in a comfortable setting, setting a purpose, obtaining the drug from a reputable source, measuring the dose and arranging a time to take the drug. Given the importance of these practices (as rated by our participants), future research should investigate why first-time users fail to employ these strategies (possibly due to lack of knowledge, the experience being more likely to be opportunistic and unplanned, or lack of autonomy over their first drug use experience) and consider how to communicate the importance of these practices to new users. In contrast, psychedelic use among experienced users appears to be well planned and this aligns with their motivations for use, with spiritual pursuits, self-enlightenment, therapeutic effects, processing of difficult emotions and artistic inspiration reported as important motivations for their most recent experience. Given these motivations, it is perhaps unsurprising that scores on the Emotional Breakthrough Inventory were higher for the most recent, as compared with the first experience. However, curiosity and fun were commonly reported motivations for both experiences (particularly so for the first experience) and this aligns with previous research which finds that pleasure is a fundamental, yet understudied motivation for and aspect of the psychedelic experience [29]. It is important to note, however, that participants’ most recent experience may not be most representative of how they typically use psychedelics, and future research may consider asking participants to consider the previous two or three experiences, or their most meaningful experience.

Participants were most likely to use psychedelics in their own home, or the home of a friend. However, use in “party” settings, such as clubs or festivals was more common for the first experience. Indeed, more participants reported ensuring they were comfortable with their surroundings (the harm reduction practice which obtained the highest score) for their most recent experience. Given the importance of set and setting for the psychedelic experience and our finding of lower Participant Harm Reduction Scores among those who took them in party settings, this gives cause for concern. While we did not see differences in the CEQ scale for first versus most recent experience, our exploratory analysis observed numerically higher scores on the CEQ when psychedelics were used in party settings, particularly for the most recent experience. Future research should explore experiences of psychedelic use (particularly among inexperienced users) in party settings, including the harm reduction practices engaged with and the extent to which these relate to positive and negative psychedelic experiences. This could inform agencies supporting psychedelics users in these settings [30].

We observed few differences in practices used during the trip between first and most recent experience. We asked participants whether they were with trusted friends and whether they had an unintoxicated trip sitter present, with relatively high endorsement of the former, but low endorsement of the latter. While psychedelic harm reduction resources often encourage having a sober trip sitter, many participants suggested this may not always be helpful. Participants suggested that simply having a sober person present, who may not have any personal experience with psychedelics, may cause issues itself, if that person is unable to understand their state of consciousness. Rather, having people present who are familiar with the psychedelic experience (such as a sober “guide”) or who are deeply trusted (but not necessarily sober) was seen as more important. Further research should investigate the role of the sober trip sitter/guide and harm reduction advice should consider recommendations which focus on the importance of being with people one trusts and who have experience with psychedelics. Our experienced users were clear that while having a support network during one’s first psychedelic experience was important, this was less important for those with more experience, with some participants reporting that they preferred to use psychedelics alone.

Participants reported greater use of harm reduction practices after the trip for their most recent compared with first experience. This was largely driven by greater engagement with creative and mindful pursuits (although whether this is due to greater engagement with these in general is unknown). Although there was little mention of these “aftercare” practices in the qualitative responses, most participants reported allowing a rest day and speaking to someone after the experience and these were the two practices with the highest Harm Reduction Score after the experience. Given other research which suggests that challenging experiences may be a fundamental prerequisite for emotional breakthrough [23] and that post hoc narratives developed through story telling with others are a critical part of processing these challenging experiences [20], future research should further examine the role of aftercare practices (such as journaling and speaking to others) in shaping psychedelic experiences. It was interesting that CEQ scores were not correlated with Harm Reduction Scores for the most recent experience (but were for the first experience), and we tentatively suggest this may be because more experienced users are better able to mitigate the effects of challenging experiences through post-trip “meaning making”, rather than relying as much on set and setting.

### Strengths, limitations and future directions

Our study is the first, to our knowledge, to examine the difference in harm reduction practices used by first time as compared with experienced psychedelic users, and therefore provides insight into which important practices might be overlooked by novice users. While all these practices were taken from respected harm reduction websites, there were some which were not deemed important either in the quantitative or the qualitative data, the effects of others were equivocal (e.g. smoking cannabis—recent research suggests that cannabis increases the intensity of the psychedelic experience, without increasing emotional breakthrough [31]), while others (e.g. having an awareness of how dose impacts the experience) were not included. Furthermore, over 70% of our participants, for both first and most recent experiences, reported engaging in at least one of the harm reduction practices we described as “myths”, with a relatively large number of participants reporting smoking cannabis, isolating themselves from others to focus on negative thoughts and problems and being in a dark room. These last two are recommended only for more experienced users, and that we saw relatively high use of these practices for participants’ first experiences is of note. Indeed, our qualitative analyses indicated that under some circumstances these practices could be beneficial or enhance the experience, particularly for more experienced users. Future research should refine our list of both harm reduction practices and myths and perform a factor analysis, as developing a list of key practices which takes into consideration user experience, has important implications for the development of drugs education. Future research should not only examine how they lead to a safe experience, but also to a positive or enjoyable one, which is important given most of our participants reported using psychedelics on both occasions for fun.

Our sample has some limitations. First, participants were recruited from forums and organisations with a special interest in psychedelics, and were largely male, which may mean their responses may not reflect those of everyone who chooses to use psychedelics. Indeed, there may be an element of “survivor bias”, such that those who remain engaged with these forums and continue to use psychedelics may have had fewer unpleasant experiences when first using psychedelics. If true, our survey may have recruited those who with a lower propensity to experiencing harm. However, our participants’ experience and engagement with psychedelic networks do mean that their ratings of the importance of the different practices may be more reliable. Our finding that greater use of practices deemed important for a safe experience was positively associated with emotional breakthrough and negatively associated with challenging experiences (at least for the first experience) should lend credibility to these ratings, but it is still possible these do not reflect the reality of the importance of these different practices and future research should examine this further. Second, we had to exclude many participants based on failure of our stringent attention check questions, and while this reduced our sample size, we see this removal of poor quality data to be a strength.

Participants’ reports of their experiences (particularly the first) may be subject to forgetting. While an individual’s first experience with a psychedelic is likely to have been salient and memorable, it is possible that harm reduction practices engaged in before or after the experience may be less likely to be recalled than those engaged in during the experience. This could have been mitigated by recruiting separate novice and experienced users, but this would have introduced differences between these groups which would make comparison between more difficult. Similarly, post-trip “meaning making”, which we tentatively suggest may be more common among experienced users, may have biased or changed participants’ memories of their experiences.

We focused on the relationship between the use of these practices and emotional breakthrough (EBI) and challenging experiences (CEQ) as proxies for positive and negative experiences. However, we know that challenging experiences may not always be negative and may be an important pre-requisite for emotional breakthrough and other positive experiences. It is possible that some subscales within the CEQ may reflect more negative experiences, and perhaps harm reduction practices might specifically reduce the likelihood of these. A new scale which assesses challenging experiences which are not later resolved may be more helpful for identifying the importance of harm reduction practices which mitigate these. We observed similar CEQ scores for the first and most recent experiences, but there may be multiple different explanations for this. First-time users may have taken a smaller dose, which would reduce their likelihood of a challenging experience. They were also more likely to report trying the psychedelic for fun, or out of curiosity, rather than for “deeper” reasons which may have been more likely to elicit challenging experiences. There are some potential limitations with our CEQ scale, as two items on the Insanity subscale were missing due to experimenter error. However, we observed good internal reliability for this scale, and our overall mean CEQ score and the Insanity subscale scores (for first and most recent experience) were similar to those observed in previous studies [32].

Our exploratory, but underpowered analyses indicated that use of harm reduction practices was lower in party settings and that use in these settings was associated with higher CEQ and lower EBI scores. Understanding how use and experiences differ in these settings and how harm reduction can mitigate possible negative effects in these settings will be important. Similarly, future research should examine the prevalence of use of different practices in different settings and how intentions influence the locations and harm reduction practices used.

## Conclusion

Our research helps us understand how use of harm reduction practices may change with experience. Our mixed methods data shed light on the perceived importance of different harm reduction practices and examine their association with the psychedelic experience itself. We find that greater use of practices deemed important for a safe experience is related to higher scores on the Emotional Breakthrough Inventory. We identify users’ motivations for use, which is important given that any harm reduction advice should be based on an understanding of why people are using the drugs in the first place. Together, our research has important implications for the development of psychedelic harm reduction advice and provides opportunities for future research to explore the importance of these different practices in more detail.

## Supplementary Information

**Additional file 1: Table S1.** Participant characteristics and psychedelic use history; **Table S2.** Characteristics of first and most recent psychedelic experiences (continued from Table 1 – details of ‘other’ responses); **Table S3.** Use of harm reduction practices and their Harm Reduction Scores

## Data Availability

Study data are available from the University of Bristol's Research Data Repository, data.bris (10.5523/bris.1r9y0tup0moyd2u03hy7jfjlgz).

## Abbreviations

CEQ: Challenging Experience Questionnaire
EBI: Emotional Breakthrough Inventory
