# Supplementary material for: Are you tripping comfortably? Investigating the relationship between harm reduction and the psychedelic experience
Source: Harm Reduct J. 2022 Jul 24;19:81. doi: 10.1186/s12954-022-00662-0 (PMC9310449; doi:10.1186/s12954-022-00662-0)
Supplement: Supplementary file 1 — Additional file 1: Table S1. Participant characteristics and psychedelic use history; Table S2. Characteristics of first and most recent psychedelic experiences (continued from Table 1 – details of ‘other’ responses); Table S3. Use of harm reduction practices and their Harm Reduction Scores [file 12954_2022_662_MOESM1_ESM.docx]

**Supplementary Materials**

**Attention checks and exclusion of participants**

There were three attention checks. The first came at the end of the demographic section and asked participants to select the option ‘maybe’. The second was midway through the harm reduction survey for their most recent psychedelic experience and participants were asked to select the option ‘yes’. The third was embedded within the harm reduction approach safety rating scale, and participants were asked to move the slider to ‘0’.

A total of 392 individuals opened the survey. Of 264 complete survey responses (many participants viewed the information sheet on the opening page and then closed the survey webpage), 82 participants were removed because they failed one of the attention check questions (n=28 check 1; n=4 check 2; n=50 check 3). The original statistical analysis plan stated that participants who responded +/-3 points from the stated anchor point for question 3 would be included. However, on closer inspection, there were a large number of responses within this range which appeared to have been completed by the same person (based on identical comments in the free text box – interestingly these all expressed negative attitudes towards psychedelics and appeared to be a form of ballot box stuffing). We therefore chose to exclude any response which was not exactly 0 for this question. A total of 12 participants were excluded because they did not complete all of the CEQ questions (an error in the survey which we identified and corrected) and a final 7 were excluded as they reported not using a classic psychedelic for one of their first or most recent experiences (first experience: salvia n=2, cannabis n=1; second experience: nitrous oxide n=1, ketamine n=2, MDMA n=1).

**Supplementary Table 1. Participant characteristics and psychedelic use history**

|  | Participants  (n=163) |
| --- | --- |
| Age (mean) | 27.50 (8.06) |
|  |  |
| Education n (%) |  |
| PhD or higher | 10 (6) |
| Masters Degree | 20 (12) |
| Bachelors Degree | 65 (40) |
| Vocational Training | 5 (3) |
| High School or Equivalent | 51 (31) |
| Some of High School | 6 (4) |
| Other | 11 (7) |
| Prefer not to say | 2 (1) |
|  |  |
| Lifetime use n (%) |  |
| LSD | 142 (87) |
| Psilocybin (magic mushrooms) | 131 (80) |
| DMT | 57 (35) |
| 2C-B | 50 (31) |
| Mescaline | 17 (10) |
| Other | 31 (19) |

*Drugs cited as ‘other’ were both those considered ‘classic psychedelics’ (e.g., 5-MeO-DMT, 25I-NBOMe, 4-HO-MET) and other drugs which can have psychedelic like effects, but are not serotonin agonists (e.g., MDMA, ketamine, Salvia divinorum, muscimol).*

**Supplementary Table 2. Characteristics of first and most recent psychedelic experiences (continued from Table 1 – details of ‘other’ responses)**

|  | First experience  (n=163) | Most recent experience (n=163) |
| --- | --- | --- |
| Drug used (‘other’ responses) n (%) | n=10 | n=30 |
| 25-I Nbome | 1 (1) | 0 (0) |
| LSA | 1 (1) | 0 (0) |
| 1P-ETH-LAD | 0 (0) | 1 (1) |
| 1cP LSD | 0 (0) | 1 (1) |
| 4-HO-MET | 0 (0) | 2 (1) |
| 5-MeO DMT | 0 (0) | 3 (2) |
| Ayahuasca | 0 (0) | 1 (1) |
| LSD + psilocybin | 4 (2) | 6 (4) |
| LSD + DMT | 0 (0) | 2 (1) |
| LSD + 2C-B | 0 (0) | 1 (1) |
| LSD + MDMA | 0 (0) | 1 (1) |
| LSD + methamphetamine | 0 (0) | 1 (1) |
| LSD + psilocybin + 2C-B | 2 (1) | 1 (1) |
| LSD + psilocybin + DMT | 0 (0) | 1 (1) |
| LSD + psilocybin + mescaline | 0 (0) | 1 (1) |
| DMT + mescaline | 1 (1) | 1 (1) |
| mescaline +MDMA | 0 (0) | 1 (1) |
| psilocybin + 2C-B | 1 (1) | 4 (2) |
| psilocybin + DMT | 0 (0) | 1 (1) |
| 2C-B + DMXE | 0 (0) | 1 (1) |
|  |  |  |
| Intention (‘other’ responses) n (%) |  |  |
| Other – shared social experience | 1 (1) | 2 (1) |
| Other – scientific interest | 1 (1) | 0 (0) |
| Other – philosophical interest | 1 (1) | 0 (0) |
| Other – personal social development | 1 (1) | 1 (1) |
| Other – process previous psychedelic experiences | 0 (0) | 3 (2) |
| Other – to introduce another person | 0 (0) | 2 (1) |
| Other – celebration | 0 (0) | 1 (1) |
| Other – part of regular habit | 0 (0) | 1 (1) |

**Supplementary Table 3.** **Use of harm reduction practices and their Harm Reduction Scores.**

|  | No. ppts reporting using this practice for first experience  (n=163) | No. ppts reporting using this practice for most recent experience  n=163) | Mean Harm Reduction Score (SD) |
| --- | --- | --- | --- |
| Before n (%) |  |  |  |
| Ensured you were familiar and  comfortable with your surroundings | 104 (64) | 132 (81) | 7.94 (3.07) |
| Measured the dose of the substance | 59 (36) | 99 (61) | 7.10 (4.07) |
| Obtained the psychedelics from a  reputable source | 78 (48) | 117 (72) | 6.75 (4.10) |
| Researched the drug and others’  experiences with the drug | 98 (60) | 89 (55) | 6.31 (4.25) |
| Tested the substances | 13 (8) | 39 (24) | 6.15 (4.61) |
| Set a goal / purpose | 35 (21) | 70 (43) | 3.19 (3.91) |
| Started with a smaller / lower dose before  consuming more | 59 (36) | 40 (25) | 3.15 (4.58) |
| Ate food (either a snack or a meal) | 89 (55) | 100 (61) | 1.18 (4.12) |
| Arranged a time to take the dose * | 86 (53) | 107 (66) |  |
| None of the above | 11 (7) | 0 (0) |  |
| Total before score |  |  | First:  17.62 (10.67)  Most recent: 23.37 (9.97) |
|  |  |  |  |
| During n (%) |  |  |  |
| Ensured you were with trusted friends | 104 (64) | 101 (62) | 6.73 (3.59) |
| Drank water or flat sugary drinks | 116 (71) | 132 (81) | 5.23 (3.75) |
| Avoided alcohol | 101 (62) | 92 (56) | 4.39 (4.42) |
| Kept note of the time you consumed the  psychedelics to track your dose | 74 (45) | 106 (65) | 4.00 (4.17) |
| Had an unintoxicated trip sitter present | 35 (21) | 31 (19) | 2.18 (4.62) |
| None of the above | 10 (6) | 4 (2) |  |
| Total during score |  |  | First:  13.02 (6.25)  Most recent:  13.90 (6.00) |
|  |  |  |  |
| During (myths) n (%) |  |  |  |
| Were in a dark room / place | 58 (36) | 61 (37) | 0.46 (4.58) |
| Drank orange juice throughout the  experience | 25 (15) | 24 (15) | 0.28 (2.71) |
| Isolated yourself from others to focus on  your own negative thoughts and problems | 37 (23) | 58 (36) | -0.09 (5.21) |
| Smoked cannabis | 63 (39) | 59 (5) | -2.50 (4.15) |
| Took Xanax or benzodiazepine | 6 (4) | 8 (5) | -2.68 (5.00) |
| None of the above | 47 (29) | 45 (28) |  |
| Total during (myths) score |  |  | First:  -0.88 (1.24)  Most recent:  -0.86 (1.32) |
|  |  |  |  |
| After n (%) |  |  |  |
| Allowed a rest day (i.e., no other  substance, rested/relaxed) | 101 (62) | 107 (66) | 5.93 (3.51) |
| Spoke about your experience with a  trusted friend or member of the  psychedelic community (online or in  person) | 98 (60) | 98 (60) | 5.07 (3.59) |
| Meditation / yoga / breathwork | 26 (16) | 68 (42) | 3.53 (4.08) |
| Engaged in a creative outlet (e.g.  journaling, painting) | 40 (25) | 64 (39) | 3.40 (4.20) |
| Allowed at least three months between  psychedelic experiences | 52 (32) | 53 (33) | 2.88 (3.85) |
| Took vitamins / supplements | 23 (14) | 46 (28) | 1.87 (3.43) |
| Used a drug charity’s online chat to  discuss your experience | 4 (2) | 8 (5) | 1.20 (3.01) |
| None of the above | 22 (13) | 13 (8) |  |
| Total after score |  |  | First:  10.31 (6.16)  Most recent:  12.26 (6.95) |

** No Harm Reduction Score for the ‘Arranged a time to take the dose’ question as this was left out of the survey in error.*

*Sources (accessed January 2021):* <https://www.release.org.uk/>; <https://www.psycareuk.org/>; <https://www.bdp.org.uk/>; <https://zendoproject.org/>; <https://psychedelicsociety.org.uk/>; <https://www.crew.scot/>
